# Extrapyramidal side effect of donepezil hydrochloride in an elderly patient: A case report

**DOI:** 10.1097/MD.0000000000019443

**Published:** 2020-03-13

**Authors:** Hong-Chun Li, Ke-Xue Luo, Jie-Sheng Wang, Qin-Xian Wang

**Affiliations:** Department of Geriatrics, Tongde Hospital of Zhejiang Province, Hangzhou, China.

**Keywords:** advanced age, donepezil hydrochloride, drug interaction, extrapyramidal reaction

## Abstract

**Introduction:**

Alzheimer disease (AD) is a neurodegenerative disease characterized by progressive cognitive dysfunction, which is mainly manifested as memory impairment and a reduced ability to self-care, often accompanied by neuropsychiatric and behavioral disorders. Donepezil is the second drug to be approved by the US FDA for the treatment of AD. Of the five FDA-approved drugs for AD treatment, donepezil is currently the most widely used. Here, we report an extrapyramidal adverse reaction to donepezil in an elderly patient with AD.

**Patient concerns:**

An 87-year-old woman presented with a 1-year history of forgetfulness that was aggravated since the past 2 months. She had a long-term history of multiple major conditions, including hypertension, diabetes, osteoporosis, and arterial plaques. Brain imaging showed age-related changes, and her Mini Mental State Examination score was 20. Other tests revealed no abnormalities apart from multiple thyroid nodules on ultrasonography.

**Diagnosis:**

She was diagnosed with AD, hypertension, type 2 diabetes mellitus, diabetic neuropathy, osteoporosis, carotid and lower-extremity arterial plaques, thyroid nodules.

**Interventions:**

She was treated with donepezil (5 mg/day), amlodipine besylate (5 mg/day), glimepiride (4 mg/day), methylcobalamin (1.5 mg/day), calcium carbonate D3 (600 mg/day), simvastatin (20 mg/day) and enteric-coated aspirin (100 mg/day).

**Outcomes:**

Four days later, she experienced fatigue, panic, sweating, and one episode of vomiting. On the 5th day, she developed increased muscle tension, speech difficulty, and involuntary tremors. Imaging and blood tests revealed no obvious abnormality, and the patient was not receiving psychotropic drugs. An extrapyramidal adverse reaction to donepezil was considered, and the drug was discontinued, after which the symptoms gradually disappeared.

**Conclusion:**

Serious adverse reactions to donepezil can occur in elderly patients, who typically require multiple medications for a variety of comorbidities. In particular, extrapyramidal reactions have occurred when donepezil is administered in combination with psychotropic drugs. However, in our patient, an extrapyramidal adverse reaction occurred in the absence of psychotropic drugs. Thus, clinicians must be aware of inter-individual differences in drug actions and possible serious adverse reactions, and carefully monitor these patients to ensure the timely detection of adverse events and their safe treatment.

## 1 Introduction

Alzheimer disease (AD) is a devastating, progressive, and irreversible neurodegenerative disorder,^[1]^ which is primarily affects the elderly.^[2]^ It is the most common type of dementia and represents 70% of all dementia cases.^[3]^ Donepezil is the second drug to be approved by the US FDA for the treatment of AD. Clinical studies in many countries have shown that this drug can improve the cognitive ability and daily life of patients with mild-to-moderate AD.^[4]^ Donepezil also ameliorates neurological and psychiatric symptoms.^[5]^ Furthermore, it effective in treating AD patients with or without cerebrovascular diseases.^[6]^ Of the five FDA-approved drugs for AD treatment, donepezil is currently the most widely used. However, some adverse reactions to donepezil use have been reported in the literature^[7,8]^ Here, we report an extrapyramidal adverse reaction to donepezil in an elderly patient with AD.

## 2 Case presentation

An 87-year-old woman was admitted to our department on March 15, 2018 due to a 1-year history of forgetfulness (described as “forgetting recent events”) that had been aggravated since the past 2 months. She had a medical history of multiple major illnesses: a 30-year history of hypertension treated with amlodipine besylate (5 mg/day), a 17-year history of type 2 diabetes mellitus treated with glimepiride (4 mg/day), a 10-year history of diabetic neuropathy treated with methylcobalamin (1.5 mg/day), a 10-year history of osteoporosis treated with calcium carbonate D3 (600 mg/day), and a history of carotid and lower-extremity arterial plaques treated with simvastatin (20 mg/day) and enteric-coated aspirin (100 mg/day). She had no history of drug or food allergies or of smoking or drinking. She had been educated up to the junior high school level.

A physical examination showed that her vitals were as follows: body temperature, 36.7°C; pulse, 65/min; respiratory rate, 19/min; and blood pressure, 127/67 mm Hg. A neurological examination revealed no nystagmus at eye level. Ultrasonography showed multiple nodules in the left and right lobes of the thyroid gland. A plain computed tomography (CT) scan of the brain showed ventricule, cistern and sulcus enlargement, but no abnormal density. Laboratory examinations revealed a fasting plasma glucose level of 6.96 mmol/L. Her Mini Mental State Examination score was 20 points. The results of other tests, such as urinalysis, and liver- and kidney-function tests, showed no obvious abnormalities. Based on the above findings, we made a diagnosis of AD associated with hypertension, type 2 diabetes with neurological diabetic complications, osteoporosis, atherosclerosis, and multiple thyroid nodules.

To her preexisting treatment plan, we added donepezil hydrochloride tablets (5 mg/day). On the fourth day after the initiation of this treatment, the patient experienced fatigue, panic, sweating, and one episode of vomiting. A random blood sugar level at this time was 7.8 mmol/L. On the fifth day, she developed general fatigue, was unable to get up, and exhibited left-sided torticollis. Her expression was indifferent; she had difficulty in speech and showed slight involuntary tremors of the upper limbs. A CT scan of the head performed at this time revealed no obvious abnormality, and blood tests showed no difference in blood electrolyte levels, and liver and kidney function. She had not experienced similar symptoms in the past, and had not taken any other medications on her own. We, therefore, considered a diagnosis of an adverse reaction to donepezil. We advised the patient to stop taking the drug, and closely monitored her for further changes. Her symptoms disappeared after donepezil treatment was stopped and have not recurred since.

## 3 Discussion

Our patient developed tremor of the upper limbs, indifferent expression, and increased muscle tension after receiving donepezil treatment for AD. Her symptoms disappeared after the drug was discontinued. Both prior to and during the symptomatic period, the patient had not taken any other drugs that could have explained these symptoms. Furthermore, no acute cerebrovascular disease or electrolyte abnormalities were found on auxiliary examinations, and the patient had not experienced similar symptoms in the past. These findings indicated that donepezil use had a temporal relationship with the adverse reaction in our patient. Therefore, according to the principle of the correlative evaluation of adverse drug reactions, we concluded that the extrapyramidal side effects in our patient were possibly caused by donepezil.

AD is a common age-related disease. With the aging of the population, the number of AD cases is increasing. According to the statistics released by the International Association for Alzheimer Disease, there were about 46.8 × 10^6^ dementia patients in the world in 2015; moreover, the number of patients with dementia has doubled every 20 years, indicating that dementia has become a serious threat to the health and quality of life of the elderly.^[9]^ The pathological features of AD include senile plaques formed by the extracellular deposition of β-amyloid, neurofibrillary tangles formed by the intracellular superphosphorylation of Tau protein, and neuron loss.^[10]^ At present, the etiology and pathogenesis of AD are not completely clear. Studies have shown that a decrease in the central neurotransmitter acetylcholine is related to the pathogenesis of AD.

Donepezil hydrochloride is a second-generation cholinesterase inhibitor that reversibly inhibits acetyl choline degradation in the brain and thereby indirectly increases the level of choline in the cerebral cortex, delaying the development of AD and vascular dementia.^[11,12]^ The drug is safe in terms of the cardiovascular health of elderly patients.^[13]^ According to the current researches, the safe dosage scale of donepezil is no more than 23 mg/day.^[14,15]^ However, excessive cholinergic action can cause gastrointestinal and neurological adverse reactions. Adverse reactions to donepezil most commonly involve the digestive system (42.86%), followed by the nervous system (22.86%).^[5]^ Extrapyramidal symptoms are rare.

Donepezil is metabolized by cytochrome P450 (CYP450) in the liver, and is mainly eliminated by the kidneys.^[16,17]^ In addition to donepezil, our patient was taking amlodipine besylate, simvastatin, and glimepiride, all of which are metabolized by CYP450. This may have led to the competitive inhibition of donepezil metabolism and increased the level of acetylcholine. Furthermore, elderly patients often have a decline in liver and kidney function. On average, the creatinine clearance rate is 40% lower in elderly people (>80 years) than in other adults.^[18]^ With the prolongation of clearance half-life, the same drug dose may lead to an increased blood drug concentration.^[18,19]^ The above reasons may have led to an adverse drug reaction caused by excessive cholinergic action.

Elderly patients often suffer from a variety of diseases and require treatment with multiple drugs at the same time. Patients with AD, in particular, often have accompanying mental problems. When AD medication is combined with psychotropic drugs, rare adverse reactions such as extrapyramidal side effects are more likely to occur due to the imbalance of acetylcholine/dopamine content. Magnuson and Liu both reported a case of an extrapyramidal adverse reaction caused by donepezil combined with risperidone.^[20,21]^ In Magnuson's reported case, the patient used donepezil 10 mg/night, the adverse event occurred within 2 weeks. In Liu reported case, the patient used donepezil 5 mg/day, the adverse event occurred after 3 days. In our patient, however, an extrapyramidal adverse reaction occurred despite the use of a conventional dosage of donepezil and without the combined use of psychotropic drugs.

Although there are some limitations in this case report, such as the absence of blood concentration of drug, it has positive significance in reminding clinicians to pay attention to the inter-individual differences and safety of drug actions in elderly patients.^[20,22]^ The present case report illustrates that rare adverse reactions, such as extrapyramidal reactions, can occur in patients receiving a conventional dosage of donepezil alone without combination with psychotropic drugs. Thus, clinicians must be aware of inter-individual differences in drug actions, carefully monitor drug use in elderly patients, and be highly alert to promptly detect adverse reactions and improve the safety of clinical medications.

## Author contributions

**Conceptualization:** Hong-Chun Li, Ke-Xue Luo, Jie-Sheng Wang, Qin-Xian Wang.

**Data curation:** Hong-Chun Li, Ke-Xue Luo, Jie-Sheng Wang, Qin-Xian Wang.

**Formal analysis:** Ke-Xue Luo, Jie-Sheng Wang.

**Funding acquisition:** Hong-Chun Li.

**Investigation:** Hong-Chun Li.

**Methodology:** Hong-Chun Li.

**Validation:** Hong-Chun Li.

**Visualization:** Jie-Sheng Wang, Qin-Xian Wang.

**Writing – original draft:** Jie-Sheng Wang, Qin-Xian Wang.

**Writing – review & editing:** Hong-Chun Li, Ke-Xue Luo.
